# Endometriosis Is Associated with Rare Copy Number Variants

**DOI:** 10.1371/journal.pone.0103968

**Published:** 2014-08-01

**Authors:** Rakesh Chettier, Kenneth Ward, Hans M. Albertsen

**Affiliations:** Juneau Biosciences, LLC, Salt Lake City, Utah, United States of America; University of Newcastle, Australia

## Abstract

Endometriosis is a complex gynecological condition that affects 6–10% of women in their reproductive years and is defined by the presence of endometrial glands and stroma outside the uterus. Twin, family, and genome-wide association (GWA) studies have confirmed a genetic role, yet only a small part of the genetic risk can be explained by SNP variation. Copy number variants (CNVs) account for a greater portion of human genetic variation than SNPs and include more recent mutations of large effect. CNVs, likely to be prominent in conditions with decreased reproductive fitness, have not previously been examined as a genetic contributor to endometriosis. Here we employ a high-density genotyping microarray in a genome-wide survey of CNVs in a case-control population that includes 2,126 surgically confirmed endometriosis cases and 17,974 population controls of European ancestry. We apply stringent quality filters to reduce the false positive rate common to many CNV-detection algorithms from 77.7% to 7.3% without noticeable reduction in the true positive rate. We detected no differences in the CNV landscape between cases and controls on the global level which showed an average of 1.92 CNVs per individual with an average size of 142.3 kb. On the local level we identify 22 CNV-regions at the nominal significance threshold (P<0.05), which is greater than the 8.15 CNV-regions expected based on permutation analysis (P<0.001). Three CNV's passed a genome-wide P-value threshold of 9.3×10^−4^; a deletion at *SGCZ* on 8p22 (P = 7.3×10^−4^, OR = 8.5, Cl = 2.3–31.7), a deletion in *MALRD1* on 10p12.31 (P = 5.6×10^−4^, OR = 14.1, Cl = 2.7–90.9), and a deletion at 11q14.1 (P = 5.7×10^−4^, OR = 33.8, Cl = 3.3–1651). Two SNPs within the 22 CNVRs show significant genotypic association with endometriosis after adjusting for multiple testing; rs758316 in *DPP6* on 7q36.2 (P = 0.0045) and rs4837864 in *ASTN2* on 9q33.1 (P = 0.0002). Together, the CNV-loci are detected in 6.9% of affected women compared to 2.1% in the general population.

## Introduction

Endometriosis is a gynecological condition that affects 6–10% of all women in their reproductive years and is defined by the presence of attached endometrial glands and stroma outside the uterine cavity [1]. The endometrial lesions remain under hormonal regulation with cyclic bleeding leading to secondary inflammation and scarring. Some endometriosis lesions become deeply invasive or metastatic; and endometriosis is considered a precursor to some types of cancer [2]. Symptoms of endometriosis include dysmenorrhea, dyspareunia, pelvic pain and infertility [1]. Diagnosis is based on clinical suspicion, clinical examination, ultrasound or magnetic resonance imaging, but can only be confirmed by laparoscopic visualization and histologic confirmation.

Endometriosis has a high degree of heritability as shown both in family and twin studies [3], [4], and genetic factors may account for as much as 51% of the latent liability [4]. Several independent genome-wide association studies (GWAS) have confirmed the involvement of genetic risk factors in endometriosis [5]–[8], however, only a small percentage of the genetic liability can be assigned to the common GWAS loci – largely leaving the genetic effect unexplained. The GWAS design is particularly well suited to evaluate common loci derived from ancestral founder events, but is not equally suited to detect rare or multiple mutations at a locus [9] one might expect in conditions with decreased reproductive fitness, such as endometriosis. Strategies complementary to SNP-based GWAS are therefore necessary to detect rare and recent genetic risk variants.

Large-scale variations in human genomic DNA sequence are commonly seen in certain regions of the genome, even in healthy individuals. Variations of at least 1 kb in length are defined as CNVs. CNVs have been reported to affect about 70% of the human genome [10], and it has been suggested that they account for more genetic variation in the genome (0.5–1%) than single nucleotide polymorphisms (SNPs), which affect about 0.1% of the genome [11]–[15]. Recently CNVs have been shown to contribute to complex diseases like autism, Crohn's disease, rheumatoid arthritis, and schizophrenia [16]–[18]. However, CNV-calling algorithms used in SNP-based CNV studies to date typically have been impeded by poor specificity (20–30%) [19]. Hence filtering methods to minimize false positives become of utmost importance for the most reliable analysis and conclusions derived from these CNVs [19].

Only one study to date has reported on the role of CNVs in endometriosis [20]. The study compared eutopic and ectopic endometrial tissue to blood among eleven endometriosis patients and found no evidence of somatic DNA copy number alterations leading to endometriosis. In the study reported here, we undertook a systematic analysis of a large case-control population to assess the role of CNVs as a genetic contributor to endometriosis. The study included the signal intensity data from our previously published endometriosis GWAS [8] to provide a high-density and comprehensive view of the CNV landscape in our endometriosis and control populations. In order to minimize false positives we applied a set of empirical filters together with a set of standard filters. To address if the CNVs we observe contribute to endometriosis we first compared the global CNV profile in the Caucasian control population to our cases including absolute CNV counts as well as the genomic length of CNVs both individually and combined; we then proceeded to identify associations to endometriosis more narrowly at the gene-level and at specific loci.

## Results

We have conducted a genome-wide CNV-study using the Illumina HumanOmniExpress high-density genotyping array. Using CRLMM [21] to read the raw signal intensity data recorded in the OmniExpress *idat*-files we summarized the *Log R Ratio* (LRR) and *B Allele Frequency* (BAF) at each SNP across all samples in the study for CNV analysis [21]–[23]. Our study population consisted of 2,126 cases and 17,974 controls after applying the LRR-SD and SNP call-rate filters, measured across 551,732 SNPs, including 18,220 X-chromosomal SNPs. Using PennCNV with a minimum window of ten probes, we identified 157,545 autosomal candidate CNVs. After applying our empirical filters the CNV-count was reduced about four-fold, to 43,560, as shown in Table 1. A random sampling of 1000 CNV plots from each of the primary-filter and post-filter stages showed a reduction in the false-positive rate from 77.7% to 13.9%. A summary of the resulting CNV-counts across the two populations are shown in Table 2. Samples with CNV-counts >6 were removed as outliers. Outliers were defined as samples with a CNV-count greater than median plus 1.5 times the inter-quartile range (IQR). After outlier removal 38,609 autosomal CNVs with a false positive rate of 7.3% remained for further analysis as shown in Table 1. The filtered CNV counts by copy-number state, before and after outlier-removal, are shown in Table 3. CNVs on the X chromosome were analyzed separately as a subset of the study population that encompasses 1,845 endometriosis cases and 6,640 female population controls. After applying all filters, we observed a total of 278 X-chromosomal CNVs in cases and 853 in controls. The Affymetrix CytoScan HD platform was used for technical replication of the CNVs identified using the Illumina Omniexpress platform. Twenty-two randomly selected cases with at least 1 CNV were used in the comparison. Among the 22 case samples we observed 54 CNVs using the CytoScan HD platform that encompassed a minimum of 10 probes on the Omniexpress chip. A comparison showed that 51 of the 54 CNVs were also detected by the Omniexpress platform resulting in a true positive rate of 94.4% for the procedure applied in this study. A list of all CNVs with position, copy-number state and case status after filtering and outlier-removal is shown in File S1.

**Table 1:** Pre and Post-filter CNV counts.

	Total CNV counts	FPR
**Raw PennCNV**	450,779 (20.96)	-
**PennCNV 10-SNP**	157,545 (7.84)	0.78
**Post-filter**	43,560 (2.17)	0.14
**Outlier-filter**	38,609 (1.92)	0.07

The table show the CNV-counts for Cases and Controls combined (n = 20,100) at four levels of filtering. First, Raw PennCNV reflect total CNV-counts initially identified by PennCNV. The second level, PennCNV 10-SNP, show the counts after applying a minimum 10-SNP window. Next, the Post-filter counts are shown, which reflect the counts after a series of empirically-derived CNV-quality filters were applied. The empirically derived criteria were set after a meticulous review of a large number of the candidate CNVs included in PennCNV 10-SNP. After grouping the Post-filter CNVs by sample it became evident that a small subset of samples (2–3%) had very high CNV-counts, and visual inspection revealed a majority of the CNVs in these samples to be false. To eliminate the excessive CNV-counts an outlier-filter was applied. The CNVs remaining after Outlier-filter were used in the association analysis. The average CNV-counts per individual are shown in parenthesis. The right-most column show the False Positive Rate (FPR) determined by visual inspection of 1,000 CNVs randomly selected at each step.

**Table 2:** Post-filter CNV counts and relative CNV frequency.

	Count by Individual		Count by CNV		Frequency of CNV	
CNV counts	CTL	ENDM	CTL	ENDM	CTL	ENDM
0	2765	299	0	0	0.154	0.141
1	4814	539	4814	539	0.268	0.254
2	4520	517	9040	1034	0.251	0.243
3	2939	350	8817	1050	0.164	0.165
4	1513	196	6052	784	0.084	0.092
5	746	95	3730	475	0.042	0.045
6	326	53	1956	318	0.018	0.025
7	138	22	966	154	0.008	0.010
8	73	12	584	96	0.004	0.006
9	40	7	360	63	0.002	0.003
10	26	9	260	90	0.001	0.004
11	14	6	154	66	0.001	0.003
12	21	5	252	60	0.001	0.002
13	7	1	91	13	0.000	0.000
14	4	1	56	14	0.000	0.000
15	2	2	30	30	0.000	0.001
16	0	0	0	0	0.000	0.000
17	3	0	51	0	0.000	0.000
18	2	1	36	18	0.000	0.000
19	3	4	57	76	0.000	0.002
20	3	2	60	40	0.000	0.001
21	0	1	0	21	0.000	0.000
22	1	1	22	22	0.000	0.000
23	1	0	23	0	0.000	0.000
24	2	2	48	48	0.000	0.001
> = 25	11	1	1049	41	0.001	0.000
Total	17974	2126	38508	5052	1.000	1.000

The CNV counts shown here represent the 43,560 candidate CNVs that remain after applying the Post-filter. The first column shows a specific CNV count. The second set of columns show the number of control and case individuals observed at each given CNV count. The center columns show the cumulative count of CNVs, and the last columns show the frequency at which a given number of CNVs are observed in each group of study-participants. A small subset of both case and control samples show highly inflated CNV counts with the highest CNV-counts being 41 and 231 in cases and controls respectively. A review of the individual CNVs in this group revealed that the vast majority of these CNVs are short (less than 20 SNPs), incorrectly called variants of the type CN = 1 and CN = 3. In fact, based on the visual inspection we generally found about 1–3 true CNVs per sample in this group. Using a systematic assessment to identify outliers we classified samples with more than 6 CNVs as outliers prone to increasingly high false-CNV counts.

**Table 3:** Filtered CNV counts stratified by copy-number state before and after outlier removal.

	Before outlier removal		After outlier removal	
CN state	ENDM	CTL	ENDM	CTL
0	5 (0.1%)	20 (0.1%)	5 (0.1%)	19 (0.1%)
1	2,501 (49.5%)	16,186 (42.0%)	1,917 (45.6%)	9,957 (47.1%)
3	2,540 (50.2%)	22,243 (57.8%)	2,273 (54.1%)	11,087 (52.5%)
4	6 (0.1%)	59 (0.2%)	5 (0.1%)	55 (0.3%)

The table summarizes the filtered CNV counts by copy-number state before and after outlier removal. A group of 77 cases (3.6%) and 351 controls (2.0%) percentage of samples were found to have very high CNV-counts (>6). Visual inspection of many of these CNVs revealed that a majority of these CNVs are false positives and that these samples generally have 1–3 true CNVs. Based on this observation we applied an outlier-removal filter to minimize the inflation of CNV-counts caused by sample specific and systematic effects. The frequency of each CN state is shown in parenthesis. After outlier removal the frequency of the different CN-states are quite similar in the case and control populations.

### Global CNV

**Figure 1:**
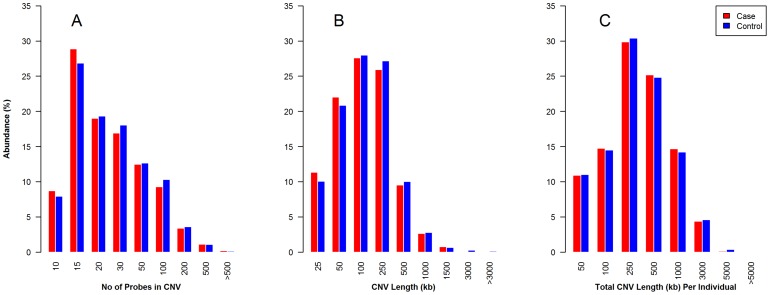
Overall comparisons of autosomal CNVs observed across the case and control populations after filtering are shown in the panels above. The data represented here reflect samples with CNV-count ≥1 after outlier-removal (cases = 1,750, controls = 14,858), autosomal probes with call-rate ≥99% (n = 533,512), and filtered CNVs (n = 38,609). Panel A show the frequency of CNVs by probe-count in various bin-sizes (10–14 probes; 15–19 probes; etc.), and Panel B show observed CNV-lengths in various bin-sizes (25 kb–49 kb; 50 kb–99 kb; etc.). The combined length of CNVs observed per individual is shown in Panel C. The case and control distributions in each panel are statistically similar implying that on a global level there is no difference between cases and controls in this study.

To investigate if endometriosis patients have excess burden of CNVs compared to the control population we compared the probe-count per CNV, the average CNV-length in kilo-bases (kb), and total length of CNVs per individual. The results of these comparisons are shown in three separate histograms in Figure 1. The results included 1,750 cases and 14,858 controls with autosomal CNV-count ≥1 after outlier removal. The overall CNV burden per individual did not differ for endometriosis cases versus controls (324 kb vs 331 kb, P = 0.48). The average CNV length per individual was slightly lower in endometriosis cases compared to controls (135 kb vs 143 kb, P = 0.027), and the average number of CNVs called per individual was similar in endometriosis cases versus controls (1.98 vs 1.91; P = 0.16 using non-parametric Mann-Whitney test). The average CNV-profiles in cases and controls are shown in Table 4. None of the three comparisons showed any measurable differences between the case and control populations implying there is no population or experimental stratification between our cases and controls. The genomic frequency plots of autosomal CNVs shown in Figure S1 in File S2 are also very similar between the two populations with characteristic spikes in the telomeric regions. The results do not support the hypothesis that excess global burden of autosomal CNVs contribute to endometriosis, but establishes that the data is free of experimental stratification. For the X-linked CNVs we found a slight enrichment of duplications in cases compared to controls (70.5% vs 61.2% respectively; P = 0.008). A summary of the distribution of large CNVs >1 Mb is shown in Table S1 in File S2 which shows that large CNVs are similar in cases and controls and have a population frequency of 2.2%.

**Table 4:** Average CNV profiles in Cases and Controls with outliers removed.

	ENDM	CTL
Probe count per CNV	32	33
Average CNV Count per Individual	1.98	1.91
Average CNV Length (kb)	135.3	143.1
Total genomic CNV (kb) per Individual	324.6	331.4

Table 4 shows the average CNV profiles in cases and controls after outlier removal. The probe count is specific to the Illumina Omniexpress platform and dependent on the SNP-filters we applied, while the CNV count and lengths are likely to reflect true population averages for CNVs about 50 kb in length or larger.

### Local CNV

**Figure 2:**
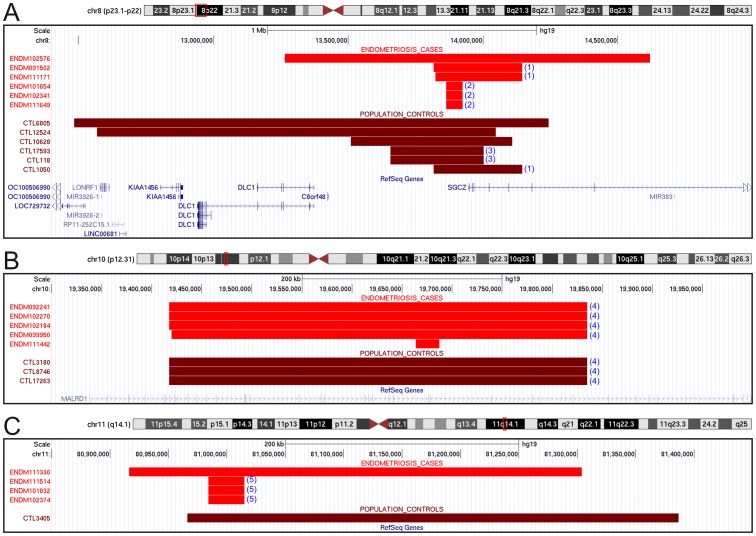
The genomic coverage of three rare copy number variant regions that show strong association with endometriosis are depicted here. The deletion at *SGCZ* on 8p22 (P = 7.3×10^−4^, OR = 8.5, Cl = 2.3–31.7) is shown in panel A, a deletion in *MALRD1* on 10p12.31 (P = 5.6×10^−4^, OR = 14.1, Cl = 2.7–90.9) is shown in panel B, and a deletion at 11q14.1 (P = 5.7×10^−4^, OR = 33.8, Cl = 3.3–1651) is shown in panel C. The genomic coverage of CNVs observed in endometriosis cases are represented in red bars and the population controls in brown bars, with genes represented in blue. The red box on each ideogram shows the chromosomal location of the CNVs. To ensure correct CNV-calls in the three regions we performed a visual inspection of the LRR and BAF plots for *all* samples in the study population. LRR and BAF plots for each of the individuals represented above are shown in Figure S1 in File S2. CNVs with apparently identical boundaries were grouped as indicated by the number in parenthesis. Haplotypes in each group were compared to determine if the CNVs in each group have shared ancestral origin.

Local CNV association with endometriosis was assessed both by gene and by CNV-segment. No gene-based associations were detected as detailed in Text S1 in File S2. For segment-based analysis the primary requirement is a genomic region shared among all CNVs at the locus, and in this analysis we found 54 non-telomeric and non-centromeric CNVRs that included at least 4 CNVs from the case population. Using ParseCNV we identified a total of 22 significant (p<0.05) CNV segments whose variation is associated with endometriosis. The associated CNVRs ranged from 15 kb to 307 kb in length. Using a multiple correction threshold of p<9.3×10^−4^ (0.05/54 CNVRs analyzed), we identified three loci strongly associated with endometriosis; a deletion in *SGCZ* on 8p22 (P = 7.3×10^−4^, OR = 8.5, Cl = 2.3–31.7), a deletion in *MALRD1* on 10p12.31 (P = 5.6×10^−4^, OR = 14.1, Cl = 2.7–90.9), and a deletion at 11q14.1 (P = 5.7×10^−4^, OR = 33.8, Cl = 3.3–1651). The three strongly associated CNVRs are listed in Table 5 together with nineteen other CNVRs that were identified at the nominal threshold (p<0.05). The genomic coverage of the samples included in the three CNVRs that pass the most conservative threshold is shown in Figure 2. High resolution CNV frequency plots of chromosomes 8, 10 and 11 are shown in Figure S2 in File S2. The LRR and BAF profiles for all 25 samples with CNVs at the three loci are shown in Figure S3, Figure S4 and Figure S5 in File S2. Twenty-one of the CNVRs listed are associated with increased risk which is concordant with aberrant function caused by disruptive CNVs, and one CNVR associated with reduced risk (protective CNVR) was observed at the nominal threshold (p<0.05). The three CNVs listed in Table 5 that are located on the X chromosome were identified using a subset of samples that included 1,845 cases and 6,640 female controls measured across 18,220 SNPs that passed quality filters. The CNVRs reported in Table 5 are the first to be linked to endometriosis. None of the 22 CNVRs listed in Table 5 are located in proximity of the previously reported GWAS associated loci. However, a review of the SNPs within each of the eighteen autosomal CNV-regions revealed two SNPs from our GWA study [8] that pass the genetic association threshold after adjusting for multiple testing using a candidate gene model as detailed in Table 6.

**Table 5:** Endometriosis CNV association results at specific loci.

CNV characteristics						Statistics		CNV Counts	
Locus	Cytoband	Gene	Probes	CNV Size (bp)	gain/loss	p-Value^a^	OR[95%CI]	Case (n = 2,126)	Control (n = 17,974)
chr3:4050668–4076977	3p26.1	*SUMF1*	13	26,309	loss	2.3×10^−2^	2.61[1.02–5.94]	8	26
chr4:186997766–187054136	4q35.1	*TLR3*	15	56,370	gain	1.0×10^−2^	6.77[1.34–31.5]	4	5
chr6:29096413–29161434	6p22.1	*OR2J2*	20	65,021	loss	3.6×10^−2^	2.03[0.98–3.88]	12	50
chr6:162828828–162864838	6q26	*PARK2*	11	36,010	loss	5.0×10^−3^	3.03[1.31–6.43]	10	28
chr7:153586831–153654045	7q36.2	*DPP6*	32	67,214	gain	2.9×10^−2^	2.99[0.96–7.96]	6	17
chr8:4231556–4261356	8p23.2	*CSMD1*	20	29,800	loss	3.1×10^−2^	4.23[0.93–15.8]	4	8
chr8:13864062–13924509	8p22	*SGCZ* b	21	60,447	loss	7.3×10^−4^	8.47[2.26–31.7]	6	6
chr9:119533737–119576802	9q33.1	*ASTN2*	21	43,065	loss	2.1×10^−2^	3.85[1.05–12.0]	5	11
chr10:14987439–15045633	10p13	*DCLRE1C*	14	58,194	gain	1.8×10^−2^	0.15[0.00–0.88]	1	56
chr10:19669367–19687397	10p12.31	*MALRD1*	9	23,660	loss	5.6×10^−4^	14.1[2.74–90.9]	5	3
chr11:80984220–81014974	11q14.1	none^c^	19	30,754	loss	5.7×10^−4^	33.84[3.3–1651]	4	1
chr11:97419744–97460206	11q22.1	none^c^	7	40,462	loss	2.2×10^−2^	4.84[1.04–19.0]	4	7
chr12:73231378–73363956	12q21.1	none^c^	13	132,578	loss	3.4×10^−3^	11.29[1.91–77.1]	4	3
chr13:20216910–20524704	13q12.11	*PSPC1-ZMYM5*	28	307,794	gain	3.5×10^−3^	7.06[1.7–27.77]	5	6
chr13:84111773–84238849	13q31.1	*SLITRK1*	27	127,076	loss	1.2×10^−2^	1.91[1.11–3.13]	20	89
chr14:63775684–63870930	14q23.2	*GPHB5-PPP2R5E*	22	95,246	gain	1.3×10^−2^	3.30[1.16–8.28]	7	18
chr19:20081948–20170214	19p12	*ZNF682*	41	88,266	gain	1.2×10^−3^	10.59[2.28–53.4]	5	4
chr19:53611187–53627882	19q13.42	*ZNF415*	12	16,695	loss	3.5×10^−3^	7.06[1.7–27.77]	5	6
chr22:42884997–42895634	22q13.2	*SERHL*	2	10,637	gain	3.1×10^−2^	4.23[0.93–15.8]	4	8
chrX:8407958–8463228	Xp22.31	*VCX3B*	5	55,270	gain	9.8×10^−3^	5.4[1.28–26.1]	6^d^	4^d^
chrX:115941080–115986516	Xq23	none^c^	13	45,436	loss	9.2×10^−3^	14.4[1.4–707.2]	4^d^	1^d^
chrX:140348507–140363732	Xq27.2	*SPANXC*	2	15,225	gain	1.3×10^−2^	2.17[1.14–4.03]	18^d^	30^d^

Copy-Number-Variant (CNV data from a case∶control cohort was analyzed for association with endometriosis. Of 34 candidate loci identified using ParseCNV 22 loci passed a nominal significance threshold upon individual inspection and three of these passed the genome-wide significance threshold of 9.3×10^−4^. The coordinates reported are based on NCBI build 37, hg19 reference sequence.

a p-Values were calculated using Fisher's exact test.

b CNV is located 20,000 bp downstream of SGCZ.

c Flanking genes over 90 kb away.

d The analysis of the X chromosome included 1,845 endometriosis cases and 6,640 female population control subjects.

**Table 6:** SNP association in CNV regions.

	Genomic position		Genotype count		Genotype frequency		SNP count		SNP association		Gene location	
SNP ID	Chr	Pos	ENDM	CTL	ENDM	CTL	CNV region	non-LD	OR	p-Value	Gene	Location
rs758316	7	153,631,648	303/1111/847	4046/11897/9468	0.378	0.393	34	7	0.94	0.0045	*DPP6*	INTRON
rs4837864	9	119,544,433	49/445/1789	300/4780/20384	0.119	0.106	22	7	1.14	0.0002	*ASTN2*	INTRON

The SNPs located within the eighteen autosomal CNV regions have previously been evaluated for association with endometriosis (Albertsen et al.), where they failed to pass the genome-wide significance threshold (p<5×10^−8^) applied in GWA studies. Using the less stringent criteria applied to candidate genes, we found that both regions have 7 non-LD SNPs (r^2^<0.2) which provide an adjusted significance threshold of 0.007. The two SNPs listed here have genotypic p-Values that are significant at this threshold. Both SNPs were in Hardy-Weinberg equilibrium (p>10^−3^).

Clinical stratification of the samples by severity (n_Severe_ = 154, n_ModerateMild_ = 1972; p = 0.56) and infertility (n_Infertility_ = 954, n_non-Infertility_ = 1,172; p = 0.59) showed no correlation to the collective CNV-burden for the CNVRs presented in Table 5. However, two of the most strongly associated CNVRs showed significant phenotypic correlation (*SGCZ* with mild endometriosis, p = 0.045; and 11q14.1 with moderated endometriosis, p = 0.014), as discussed below.

A search for very rare disease-associated CNVRs revealed 13 distinct CNV regions with ≥2 CNVs in cases and none seen among the 17,974 Caucasian controls and shown in Table S2 in File S2. Ten of the thirteen CNVs overlapped the boundaries of known genes. The very rare, intergenic CNV-region on chromosome 1 includes three deletions and one duplication, which, if the duplication is considered to have a deleterious effect is very strongly associated (P = 1.24×10^−4^). Among the genes affected by the very rare CNVs are *FOXO3*, which recently has been shown to be differentially methylated and differentially expressed in a comparison between normal endometrial and endometriotic stromal cells [24], and TFF3, which is an estrogen-responsive gene shown to be up-regulated in endometrioid adenocarcinomas [25].

### Shared Haplotypes

A review of the CNVs presented in Figure 2 show several CNVs with similar start and end points. In fact, we observe five groups of samples identified in Figure 2 as 1, 2, 3, 4 and 5 with two or more apparently identical CNVs. To determine if the samples within each group share a single ancestral CNV-event, or whether each CNV has independent origins, we phased the genotypes of those samples using IMPUTE2 (as described in Materials and Methods) and then compared the resulting haplotypes in the 100 kb regions flanking the CNVs. Among the five groups investigated; 2, 3, 4 and 5 each share a rare haplotype that is significantly enriched compared to the general population (p = 2.4×10^−7^, p = 3.9×10^−5^, p = 5.7×10^−8^ and p = 6.5×10^−6^; Fisher's exact test).

## Discussion

To search for genetic risk factors in endometriosis we have previously conducted a large two-stage GWAS where we employed the Illumina HumanOmniExpress high-density genotyping array (Illumina; San Diego, CA). In addition to producing genotypes, the array also provides the opportunity to assess allelic copy number variations at each locus by evaluating the LRR and BAF for each allele across all SNPs on this array. Only samples of European ancestry with a genotyping call-rate ≥99% were considered in the analysis. Using computer-based CNV calling algorithms has been essential to our study, but while the false-negative rate is minimal we found the false-positive rate to be very high. This caused us to implement a series of restrictive quality filters like LRR SD, SNP call-rate, and minimum CNV probe-window to arrive at the CNVs we included in our statistical analysis. After applying the filters our study population consisted of 2,126 cases and 17,974 controls resulting in 157,454 CNV counts. However, visual inspection of a substantial number of these CNVs revealed various patterns characteristic of false CNVs that were readily distinguishable (Figure S6 and Table S3 in File S2). On the basis of our observations we devised a series of additional filters to further reduce the number of false positive CNVs (see Materials and Methods). As shown in Table 1, the CNV-count was reduced about four-fold, to 43,560, after applying the empirical filters. Yet, Table 2 also reveal that a small percentage of samples have very high CNV counts (>10). Visual inspection of the CNVs in many samples with high CNV-counts found the majority of these CNVs to be artefacts and that the count of true CNVs in these samples typically ranged between one and three. To provide a systematic assessment we performed an outlier removal procedure which resulted in 38,609 CNVs (shown in Table 1). The results of these efforts were a reduction in the false positive rate from 77.3% to 7.3% with a concordance rate between technical platforms of 94.4%. The final CNV data show very high correlation between the endometriosis and control populations and suggest excellent technical and biological concordance as illustrated in Figure 1.

We then proceeded to test the hypothesis that excess global burden of CNVs contribute to endometriosis, but neither the individual CNV-counts nor the individual or combined CNV-lengths showed statistical differences between cases and controls and we conclude that there is no difference between the groups on a global level. Similarly, we analyzed the data for support of the hypotheses that excess large CNVs, and gene-based CNVs contribute to endometriosis but again we found no support (see also Table S1 in File S2). Likewise there was no support for CNV association among 8 genes previously associated with endometriosis by SNP association studies.

Lastly we tested the hypothesis that locus-specific CNVs contribute to endometriosis. In this analysis we identified three CNVs on chromosome 8p22, 10p12 and 19p12 at a conservative genome-wide significance threshold (p<9.3×10^−4^) together with nineteen other CNVs that are nominally associated with endometriosis. This is significantly higher than the 8.15 randomly associated CNVRs expected based on case-status permutation (p<0.001). Of the 22 associated CNV we found 21 to be associated with increased risk. A review of the SNPs within the 19 autosomal CNVRs from our previously published GWAS [8] revealed two SNPs (rs758316 and rs4837864; Table 6) that passed the regional significance threshold for association. The apparent lack of overlap between association signals identified by SNPs and CNVs illustrate the importance of applying complementary techniques to identify the distinct genetic mechanisms that contribute to complex disease

The most significant CNVR we found is a deletion and that spans 24 kb within *MAM and LDL receptor class A domain containing 1* (*MALRD1*) on chromosome 10. *MALRD1* has been known in mouse and was inferred in man only recently but little is known about its function. The second most significant CNVR is located in a 2 Mb gene desert on chromosome 11. Since there is no gene in the vicinity the locus must be presumed to have structural or regulatory function.

The third most significant CNVR is located immediately downstream of a known gene called ζ-sarcoglycan (*SGCZ*). *SGCZ* is one of six members of the sarcoglycan gene-family that combine to form two different quarto-meric sarcoglycan complexes; one found in striated muscles that include *SGCA*, *SGCB*, *SGCD* and *SGCG*, and the other which is found in smooth muscles and include *SGCB*, *SGCD*, *SGCE* and *SGCZ*. Smooth muscle structures have recently been identified in endometroid lesions [26] establishing a plausible functional link to the involvement of *SGCZ* in endometriosis. Further, CNVs in both *SGCZ* and *PARK2* (Table 4) have also been associated with obesity-related traits in African Americans [27] suggesting that these two genes function in a concerted manner. *PARK2* is part of a region on chromosome 6 known as *FRA6E*. This region is known as a fragile area because it is unstable and prone to breakage and rearrangement. Changes involving the *FRA6E* region have been reported in several forms of human cancer, including glioblastoma, colorectal cancer, lung cancer, and ovarian cancer.

Haplotype analysis of the CNVs shown in Figure 2 suggests that CNVs in groups 2, 3, 4 and 5 were derived from shared ancestral events. To explore the genetic relationship between these individuals, we performed the identity-by-decent (IBD) between individuals in each group. None of them showed any evidence of IBD (8 or less meiosis), suggesting that these CNVs arose in shared ancestral events 5 or more generations ago. We could not determine with certainty if group 1 also was a consequence of an old ancestral event since they shared a common haplotype (p>0.05).

We made a striking observation pertaining to the phenotypic disease classification of two of the most strongly associated CNVRs. All six case samples with CNVs at the *SGCZ* locus were classified as mild, superficial disease in the *cul-de-sac*, and no ovarian involvement. In contrast, the four samples with CNVs at the chr11q14.1 locus all presented with moderate disease and ovarian endometrioma. This supports the view that disease heterogeneity might be governed by distinct mechanisms.

In a separate observation, not related to endometriosis, we note that large rare CNVs were enriched in duplications in the general population (Table S1 in File S2). This suggests that large deletions generally are more detrimental than large duplications. We also found significant enrichment of duplications on the X chromosome compared to the autosomes in our combined population (63.8% vs. 56.1%; p = 2.2×10^−7^) implying that deletions on the X chromosome are under increased selective pressure in males.

Our study has sufficient power (>80%) to detect CNV association at or above 0.1% frequency with an effect size (OR) >2.5 (Table S4 in File S2). However, we do note that one limitation of this study pertains to the limited resolution of CNVs inherent to the technical platform. For that reason we cannot make any firm conclusions regarding CNVs in the 1–50 kb range.

At least one of the 21 risk-associated CNVR is present in 6.9% of the endometriosis cases as opposed to 1.8% of the controls which suggests that CNVs are likely be important markers of endometriosis. This study demonstrates that CNVs are likely to play an important role in endometriosis. However, the study also emphasizes the critical importance of applying stringent quality filters to the raw CNVs, followed by visual inspection of associated CNVs. The CNVs we report here have not previously been associated with endometriosis and represent regions of special interest for independent replication studies. If confirmed, the CNVs described here will provide a significant contribution to the understanding of the genetic risk and pathogenesis of endometriosis.

## Subjects and Methods

### Ethics Statement

All subjects and controls provided written informed consent in accordance with study protocols approved by Quorum Review IRB (Seattle, WA 98101).

### Participant Recruitment and Medical Review

Patients included in the present study were invited to participate via an outreach program at www.endtoendo.com, where our research initiative is described in more detail. Briefly, the “End to Endo” website provides general information regarding endometriosis and our research objective, and invites women diagnosed with endometriosis to participate in our study. The inclusion criterion in the endometriosis case population in the present study is surgically confirmed diagnosis of endometriosis. Clinicians performed the medical record review and clinical assessment and patients were grouped into one of three classes of severity: mild, moderate or severe, following the general guidelines set forth by ASRM [28]. Control samples of European ancestry were collected from the general population in a separate effort, and included 10,740 females and 7,234 males. Controls did not undergo medical review and may be expected to have a 6–10% incidence of endometriosis among females, and a similar carrier-burden in males.

### DNA Extraction, Microarray Genotyping and Technical Replication

DNA was extracted from saliva samples collected using the Oragene 250 saliva collection kit (DNA Genotek; Ottawa, Ontario, Canada). DNA was extracted using an automated extraction instrument, AutoPure LS (Qiagen; Valencia, CA), and samples were genotyped using the Illumina Human OmniExpress Chip (Illumina; San Diego, CA) according to protocols provided by the manufacture's. Technical replication was performed using the Affymetrix CytoScan HD chip according to the manufacturer's protocol (Affymetrix; Santa Clara, CA).

### Assessment of Ethnicity

ADMIXTURE (ver. 1.22) [29] was used to estimate the individual ancestry proportion based on three *a priori* defined ancestry groups: European, Asian and African that were defined based on the POPRES dataset [30]. Our ancestry analysis was based on the 33,067 SNPs with the greatest ancestral frequency variation as determined by Fixation Index (***F***_ST_), that were present on both the Human Omniexpress chip used in this study, and the Affymetrix 5.0 chip used in the original POPRES experiment. To be considered for the present study the admixture proportion of a given sample was required to be ≥95% European.

### Sample and SNP Inclusion Criteria

Our study included 2,126 surgically confirmed endometriosis patients and 17,974 population controls selected from an initial pool of 2,322 cases and 19,186 controls previously determined by ADMIXTURE be ≥95% European. DNA samples from the initial pool were excluded from the study if they missed any of the following criteria: a) evidence that a sample was more closely related to another participant than 3^rd^-degree (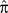
≥0.20) as determined by the **–genome** option implemented in PLINK [31], b) per sample genotyping call-rate <98%; or c) self-reported gender different from genotypic gender. A set of 533,512 SNPs of the initial 730,000 SNPs present on the OmniExpress genotyping array passed both inclusion filters of a) being autosomal, and b) having a SNP call-rate of ≥99%. SNPs on the X chromosome were analyzed separately and included 1,845 cases and 6,640 female controls measured across 18,055 SNPs that passed the sample and SNP criteria mentioned above.

### CNV Tools and Analysis

The R package CRLMM [32] was used to process raw microarray data contained in the Illumina HumanOmniExpress *idat*-files into copy-number-relevant data. The data was processed in batches of 96 Well DNA plates to extract the log R ratio (LRR: measure of total intensity of probes) and B allele frequency (BAF: measure of relative intensity of allelic probes) for each SNP. PennCNV [22], which is based on a Hidden Markov Model (HMM), was used to identify CNVs and determine CNV states (CN = 0,1,2,3,4 copy number) for each SNP based on the LRR and BAF values.

Stringent quality controls were applied to eliminate poor quality samples and false positive CNV calls. The quality threshold metric used for our microarray dataset were a) SNP call rate >99%, b) Standard deviation of allelic intensity SD LRR<0.24 as recommended [33], c) G/C base content waviness factor (GCWF) <0.05, and d) removal of CNVs spanning less than 10 SNPs. ParseCNV [33], an integrative copy number variation association software that takes CNV calls and creates probe-based statistics for individual CNVs and CNV-regions (CNVR) in case-control designs, was used to analyze and annotate CNV calls. CNV carrier frequencies between endometriosis subjects and controls were compared using one-sided Fisher's exact test. A nominal p<0.05 was considered significant. The probe-based statistic output is then merged into CNVRs based on probe proximity (<1 MB) and comparable significance (+/−1log p-value) of neighboring probes as calculated using ParseCNV. The CNVRs were further restricted to the shared overlap of all CNVs in the region. Finally, the case enriched CNVRs were filtered based on following criteria: a) a minimum of 4 CNV calls had to be present in the case-population to assure reasonable statistical power for the association analysis, and b) CNVs had to not overlap with telomeric or centromeric regions based on ParseCNV annotation. All coordinates reported are based on NCBI build 37, hg19 reference sequence. Veracity of all the associated CNVRs was visually confirmed using the LRR and BAF plots.

### Post CNV-Call Filtering

A set of post CNV-calling filtering metrics were derived empirically to reduce false CNV calls from the analysis. Visual inspection of a substantial subset of these CNVs (>10,000) revealed various patterns characteristic of false CNVs readily distinguishable to the human eye (see examples in Figure S6 in File S2). On the basis of our observations we devised a series of post CNV-calling filtering metrics referred to as “post-filter”. The filters apply to 150 kb on either side of the CNV and assess the strength of shifts in LRR and BAF values across a CNV. For LRR, the absolute mean LRR difference between the CNV region and the flanking region were utilized to determine the shift in LRR values. For single deletion absolute LRR differences between the CNV region and the upstream and downstream flanking region >0.35 was categorized as valid CNVs, while for duplications the absolute LRR difference was required to be at least 0.125 to label them as valid CNVs. For BAF, the K-means clustering algorithm was used to identify the optimal cluster to categorize into the different copy number states. We used the sum of squared error (SSE) to identify the optimal cluster size for clusters ranging from 1 to 5. For deletions, CNVs with two optimal clusters were deemed valid, while for duplication CNVs with 4 or 5 optimal clusters were deemed valid. All homozygous deletions (CN = 0) and double duplications (CN = 4) were visually inspected to confirm CNV state.

### Gene-Based CNV Analysis

All CNVs within gene-boundaries were used to assess the possible enrichment of gene-based CNVs in cases compared to controls. The cumulative burden of CNVs both rare and common can be effectively combined on a gene level using this approach. To test for genes with CNVs associated with endometriosis, we used one-sided Fisher's exact test to test for enrichment of either deletion or duplication CNVs overlapping genes between endometriosis subjects and controls. A total of 82 genes with 4 or more CNVs were evaluated. Accordingly, a multiple correction threshold of p<6.0×10^−4^ was used to determine significance. The exact boundaries of the known genes were used based on NCBI build 37, hg19 reference sequence.

### Large CNV Analysis

For large CNV analysis, CNVs were considered only if they had a length >1 Mb. To test the hypothesis that excess large CNVs contribute to the risk of endometriosis, we used one-sided Fisher's exact test to test for burden of excess large CNVs in endometriosis subjects compared to healthy controls. The burden analysis was performed for both deletions and duplications separately. A p<0.05 was considered statistically significant.

### Phasing and IBD Analysis

IMPUTE2 (ver. 2.2.2) was used for phasing the typed genotypes. We performed the phasing under the following settings: k = 80 (tuning parameter for phasing updates), iter = 30 (total number of MCMC iterations), burnin = 10 (number of iter to discard as burn-in) and Ne = 20000. The Ne parameter represents the effective size of the population being analyzed, and it is used to scale the recombination rates in the imputation HMM. The whole genome was split into 5 Mb non-overlapping regions using a 250 kb buffer size to prevent the edge effects on either side (default in IMPUTE2). The resulting two haplotypes for each individual were aligned across chunks using the phase of heterozygous genotypes near the center of the overlap region, and the chunks were merged to yield chromosome-wide phasing. The phased genotypes were then analyzed using GERMLINE for discovering long shared segments of Identity by Descent (IBD) between pairs of individuals. In order to minimize the effect of IBS (Identity by State) or high LD segments, we performed LD pruning (r^2^<0.8) prior to using GERMLINE. GERMLINE was performed with the following parameters: minimum length for match (5 MB), maximum number of mismatching homozygous markers (0) and maximum number of mismatching heterozygous markers (0).

### Power Analysis

Power calculations were performed using QUANTO (ver. 1.2), using a log-additive model. The analysis included 2,126 cases and 17,974 controls with the following assumptions: Type I error = 0.05, a minor allele frequency ≥0.001 and the odds-ratio ≥2.5.

### Web Resources

End to Endo, Juneau Biosciences patient outreach program: http://www.endtoendo.com

USCS Genome Browser, http://genome.ucsc.edu

The R Project for Statistical Computing, http://www.r-project.org

CRLMM, http://www.bioconductor.org/packages/release/bioc/html/crlmm.html

PennCNV, http://www.openbioinformatics.org/penncnv

ParseCNV (ver. 1.2), http://parsecnv.sourceforge.net

ADMIXTURE (ver. 1.22), http://www.genetics.ucla.edu/software/admixture

PLINK (ver 1.07), http://pngu.mgh.harvard.edu/~purcell/plink

QUANTO (ver. 1.2.4), http://hydra.usc.edu/gxe

PubMed, http://www.ncbi.nlm.nih.gov/pubmed

IMPUTE2, https://mathgen.stats.ox.ac.uk/impute/impute_v2.html

GERMLINE, http://www1.cs.columbia.edu/~gusev/germline

## Supporting Information

File S1 Includes **Dataset S1** The dataset includes the genomic location, copy-number state and case-status of all CNVs after filtering and outlier-removal.
(TXT)

File S2 Includes **Text S1**. Gene-based analysis. **Figure S1** Frequency plots of CNVs in 17,974 population controls (Panel A) and 2,126 endometriosis cases (Panel B) as a function of genomic position. The frequency is determined on the combined deletion and duplication counts. Chromosomes are colored blue and black alternatingly. The panels show strong concordance in between controls and cases as seen at the telomeres of chromosomes 1, 6, 12 and 22. **Figure S2** High-resolution frequency plots of CNVs on chromosomes 8, 10 and 11. The genomic position in million base-pairs (Mbp) is given on the X-axis and the absolute counts are given on the Y-axis. Duplications are shown in blue and deletions are shown in red, and triangles indicate counts above 120. Dashed vertical lines indicate the positions of CNVs associated with endometriosis identified in the present study. **Figure S3** The figure shows the LRR and BAF plots of all samples with a CNV at the *SGCZ* locus. Cases have individual IDs starting with ENDM and population controls have IDs starting with CTL. **Figure S4** The figure shows the LRR and BAF plots of all samples with a CNV at the *MARLD1* locus. Cases have individual IDs starting with ENDM and population controls have IDs starting with CTL. **Figure S5** The figure shows the LRR and BAF plots of all samples with a CNV at the chromosome 11q14.1 locus. Cases have individual IDs starting with ENDM and population controls have IDs starting with CTL. **Figure S6** The figure shows in the left-side panel the characteristic plots of the four different CNV-states discussed in the paper. Each copy-number state has certain characteristics that define a correct call and, conversely, there are characteristics that are incompatible with a given CN-state (summarized in Table S3 in File S1). The three examples of incorrectly called CN = 0 all show inadequate LRR-shift and narrow bands around 0.5 in the BAF panel suggest the samples are heterozygote. The incorrectly called CN = 1 show that the LRRs in the CNV regions to be generally similar to the flanking segments and the BAF in the middle sample has signal around 0.5 suggesting the sample is a heterozygote. In the case of the incorrectly called CN = 3 examples the LRR show inadequate shift relative to the flanking segments and third of the samples show BAF signal around 0.5 which is incompatible with this state. As for CN = 4 the LRR in the CNV regions similar to the flanking segments and none of the BAF patterns has the characteristic 5-band profile. **Table S1** The table shows the absolute counts of CNVs>1 Mb with the corresponding population frequency reported in parenthesis. The results indicate that CNVs>1 Mb represent 1.1% (442 of 38,609) of all CNVs post-filter in the present study, and have a population prevalence of 2.2% (442 of 20,146). There is a slight enrichment of large CNVs in controls compared to cases, indicating a lack of association between large CNVs and endometriosis. Interestingly the proportion between deletions (31%) and duplication (69%) in these large CNVs differ significantly from the overall proportion between deletions (47%) and duplications (53%). One interpretation of this phenomenon could be that large deletions are more detrimental compared to duplications. Large CNVs are frequently reported by PennCNV as multiple adjacent CNVs. This is due to local fluctuations in LRR and BAF which causes such large CNVs (predominantly duplications) to break apart. To assess the true extend of large CNVs we merged adjacent duplications from the same individual that were less than 100 kb apart. We found 1419 CNVs that after merging were collapsed into 667 larger CNVs. Only the 442 CNVs>1 Mb are included here. **Table S2** The table list 13 CNVRs with at least 2 CNVs found in cases only. The intragenic CNVR on 1q31.3 include three CNVs with loss and one with gain. If all four CNVs have deleterious effects this CNVR also pass the genome-wide threshold for significance. **Table S3** Based on the characteristics provided in the table we devised a set of filters to apply to the raw PennCNV CNV calls. After applying these filters a large portion of the incorrectly assigned CNV calls were eliminated. **Table S4** The Power-to-Detect CNV association in the present study of 2,126 endometriosis cases and 17,974 population controls is shown given a range of odds-ratios (OR) between 2 and 5 and CNV frequencies ranging between 0.001 and 0.005.
(DOCX)
