# Supplementary material for: Endometriosis Is Associated with Rare Copy Number Variants
Source: PLoS One. 2014 Aug 1;9(8):e103968. doi: 10.1371/journal.pone.0103968 (PMC4118997; doi:10.1371/journal.pone.0103968)
Supplement: File S2 — Includes Text S1. Gene-based analysis. Figure S1 Frequency plots of CNVs in 17,974 population controls (Panel A) and 2,126 endometriosis cases (Panel B) as a function of genomic position. The frequency is determined on the combined deletion and duplication counts. Chromosomes are colored blue and black alternatingly. The panels show strong concordance in between controls and cases as seen at the telomeres of chromosomes 1, 6, 12 and 22. Figure S2 High-resolution frequency plots of CNVs on chromosomes 8, 10 and 11. The genomic position in million base-pairs (Mbp) is given on the X-axis and the absolute counts are given on the Y-axis. Duplications are shown in blue and deletions are shown in red, and triangles indicate counts above 120. Dashed vertical lines indicate the positions of CNVs associated with endometriosis identified in the present study. Figure S3 The figure shows the LRR and BAF plots of all samples with a CNV at the SGCZ locus. Cases have individual IDs starting with ENDM and population controls have IDs starting with CTL. Figure S4 The figure shows the LRR and BAF plots of all samples with a CNV at the MARLD1 locus. Cases have individual IDs starting with ENDM and population controls have IDs starting with CTL. Figure S5 The figure shows the LRR and BAF plots of all samples with a CNV at the chromosome 11q14.1 locus. Cases have individual IDs starting with ENDM and population controls have IDs starting with CTL. Figure S6 The figure shows in the left-side panel the characteristic plots of the four different CNV-states discussed in the paper. Each copy-number state has certain characteristics that define a correct call and, conversely, there are characteristics that are incompatible with a given CN-state (summarized in Table S3 in File S1). The three examples of incorrectly called CN = 0 all show inadequate LRR-shift and narrow bands around 0.5 in the BAF panel suggest the samples are heterozygote. The incorrectly called CN = 1 show that the LRRs in the [file pone.0103968.s002.docx]

SUPPLEMENTAL DATA

File S2

Endometriosis is Associated with Rare Copy Number Variants

File S2 contains 1 Text, 6 Figures and 4 Tables.

**Text S1**. Gene-based analysis. To search for gene-based CNV enrichment we considered all CNVs that had some overlap within the genomic start and end boundaries of a gene, and considered loss and gain variants together and separately. Using a genome-wide gene-based enrichment analysis, a total of 5250 genes showed at least a single overlapping CNV within case or control. We further restricted the analysis to non-telomeric and non-centromeric genes with at least 4 CNVs in the case population after which 82 genes passed the criteria. After adjusting for multiple testing (p<6×10^-4^), we did not find any gene based CNV associated with endometriosis. We specifically examined CNVs in endometriosis genes previously reported by SNP-based GWAS including: WNT4, FN1, GREB1, ID4, IL33,CDKN2BAS, HNRNPA3P1 and VETZ [[5-8](#_ENREF_5),[22](#_ENREF_22)], but none of them showed any CNV enrichment with endometriosis even at the nominal p-value threshold.


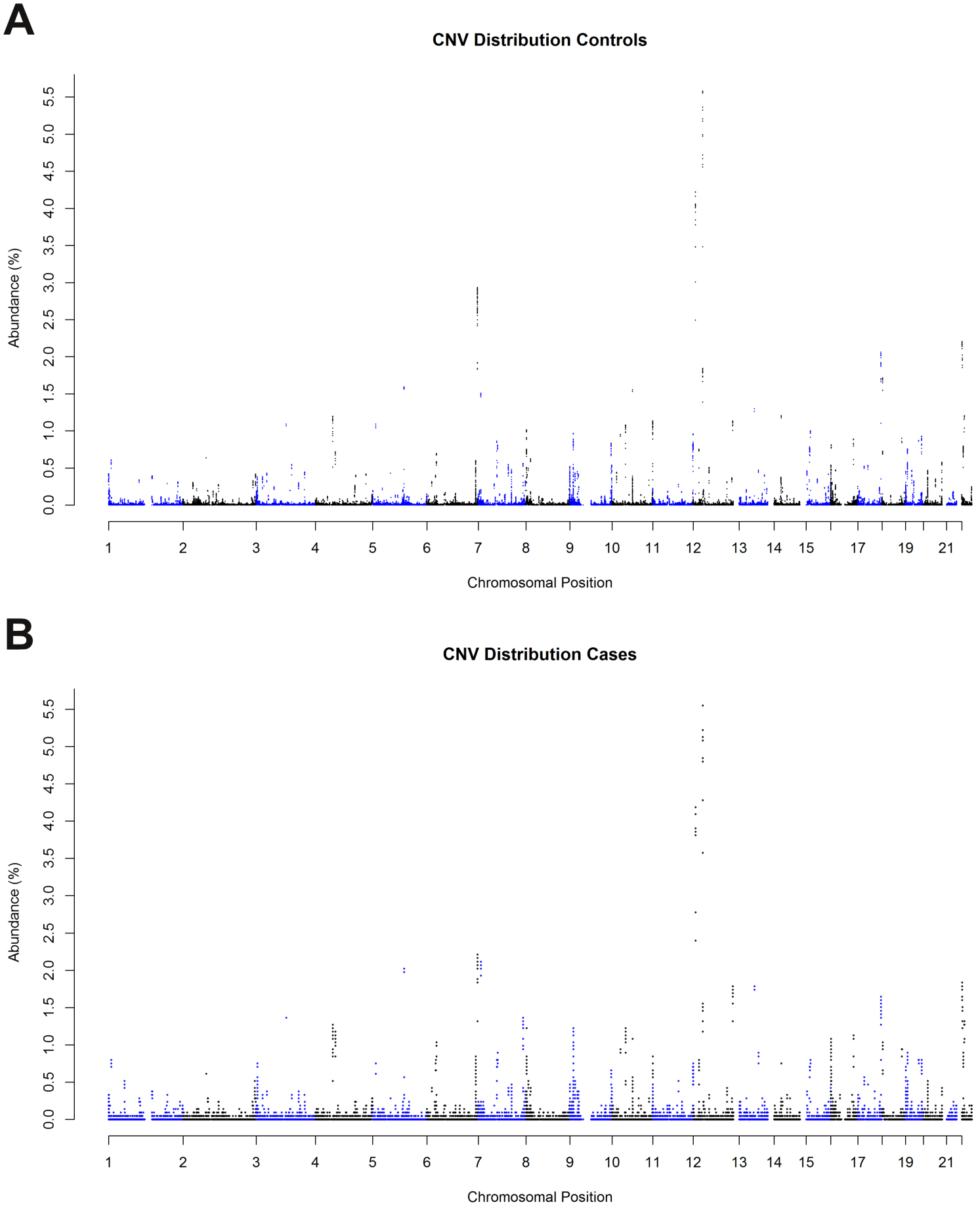


**Figure S1**. Frequency plots of CNVs in 17,974 population controls (Panel A) and 2,126 endometriosis cases (Panel B) as a function of genomic position. The frequency is determined on the combined deletion and duplication counts. Chromosomes are colored blue and black alternatingly. The panels show strong concordance in between controls and cases as seen at the telomeres of chromosomes 1, 6, 12 and 22.


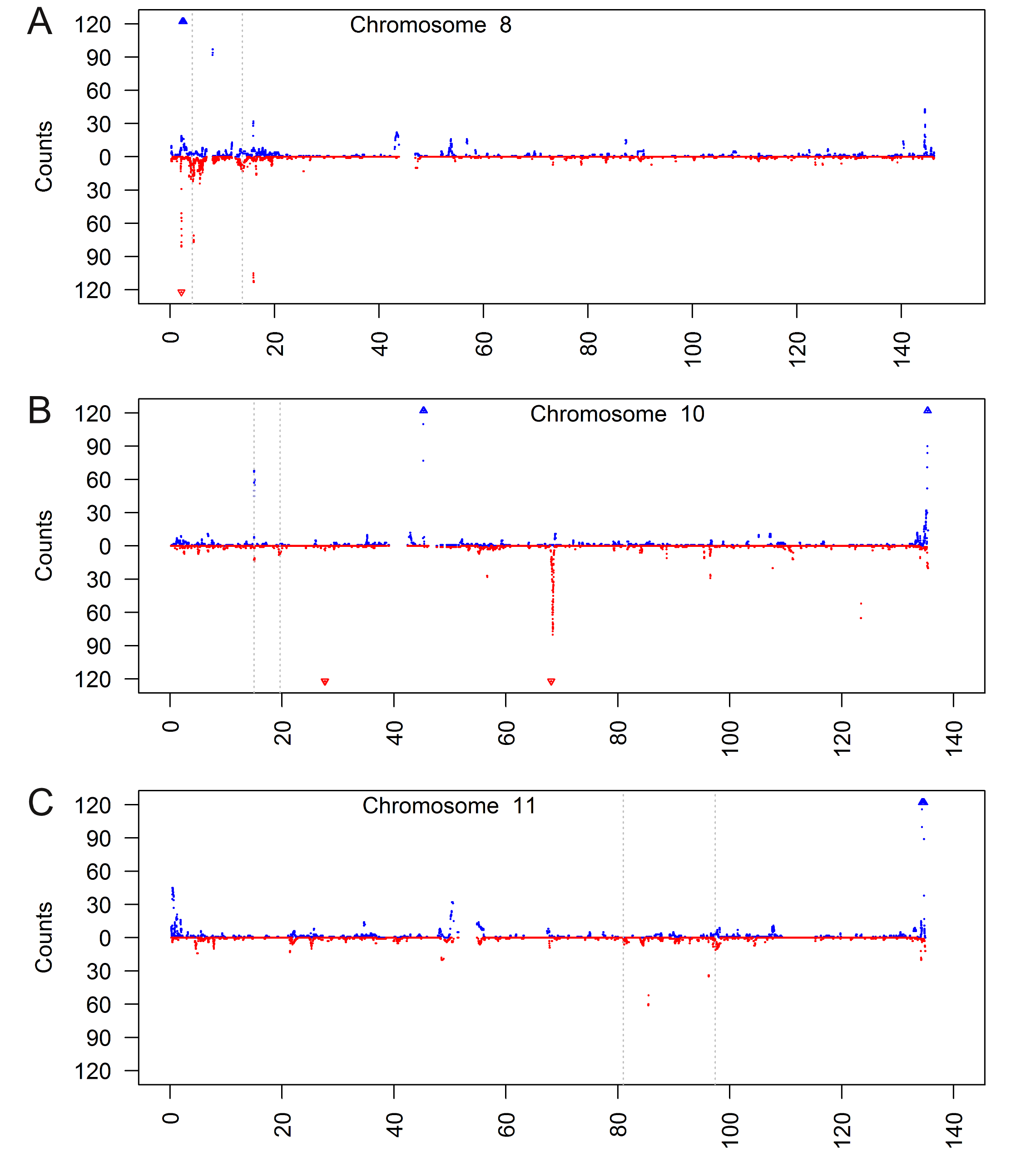


**Figure S2**. High-resolution frequency plots of CNVs on chromosomes 8, 10 and 11. The genomic position in million base-pairs (Mbp) is given on the X-axis and the absolute counts are given on the Y-axis. Duplications are shown in blue and deletions are shown in red, and triangles indicate counts above 120. Dashed vertical lines indicate the positions of CNVs associated with endometriosis identified in the present study.


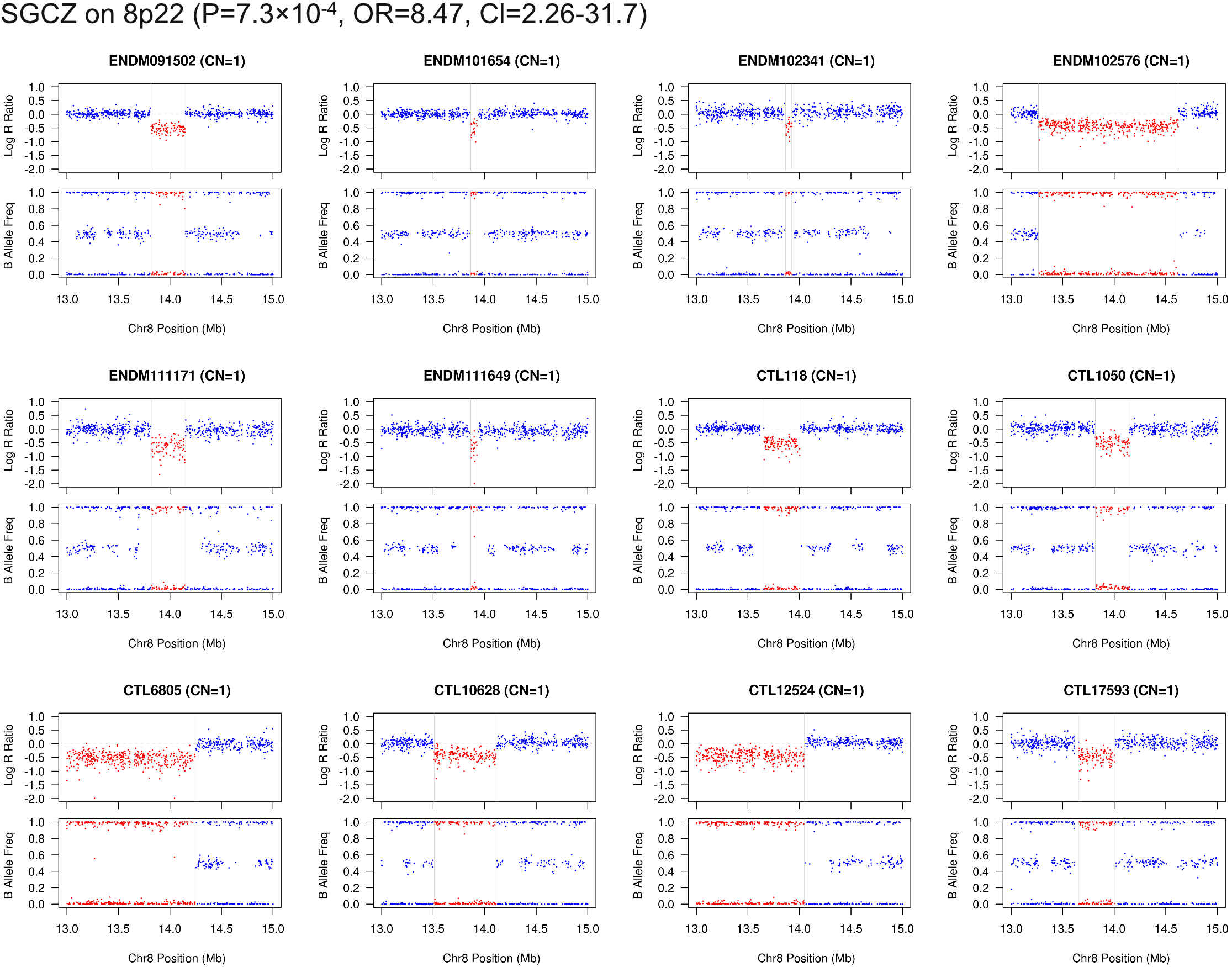


**Figure S3**. The figure shows the LRR and BAF plots of all samples with a CNV at the SGCZ locus. Cases have individual IDs starting with ENDM and population controls have IDs starting with CTL.


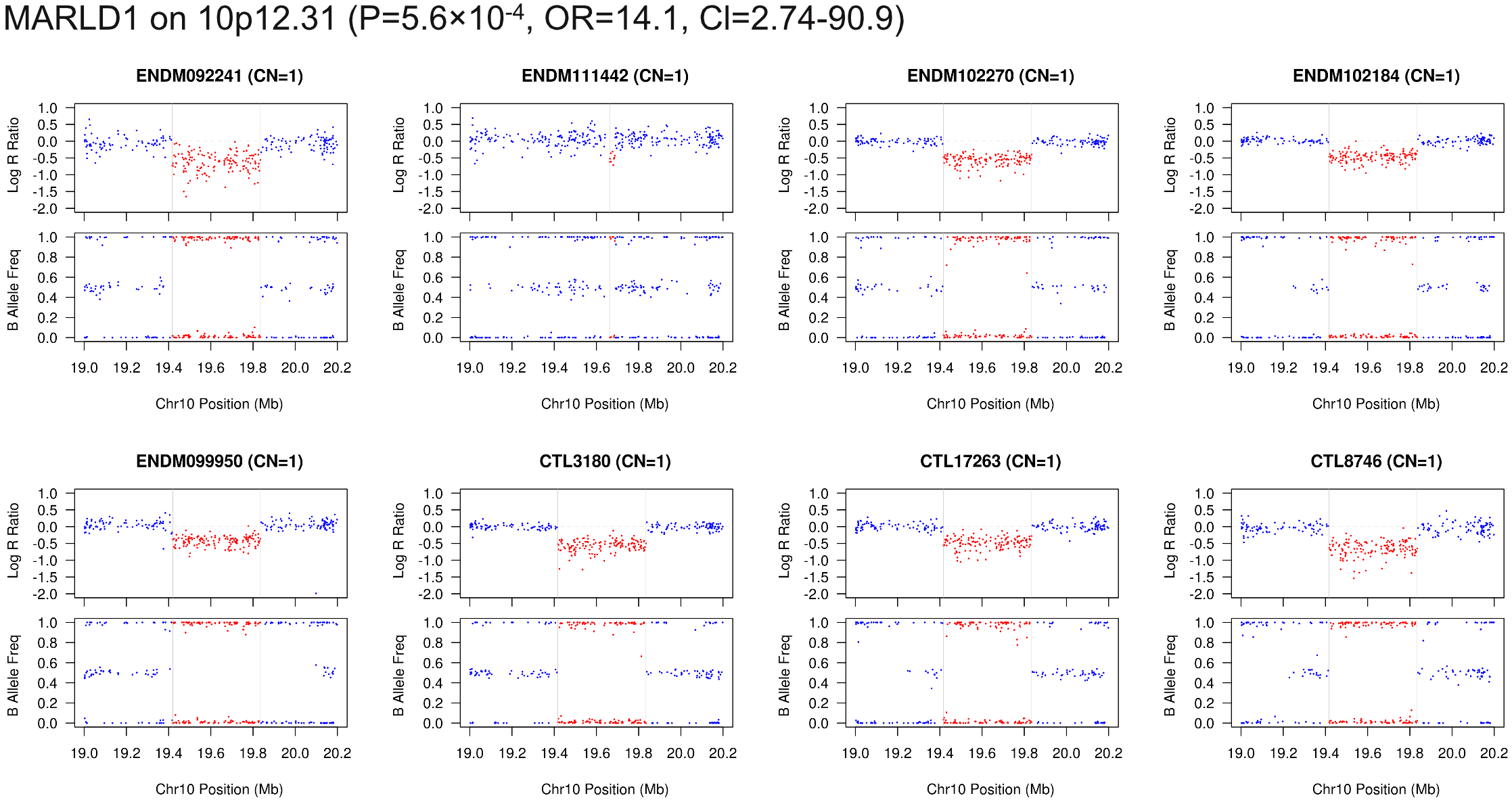


**Figure S4**. The figure shows the LRR and BAF plots of all samples with a CNV at the MARLD1 locus. Cases have individual IDs starting with ENDM and population controls have IDs starting with CTL.


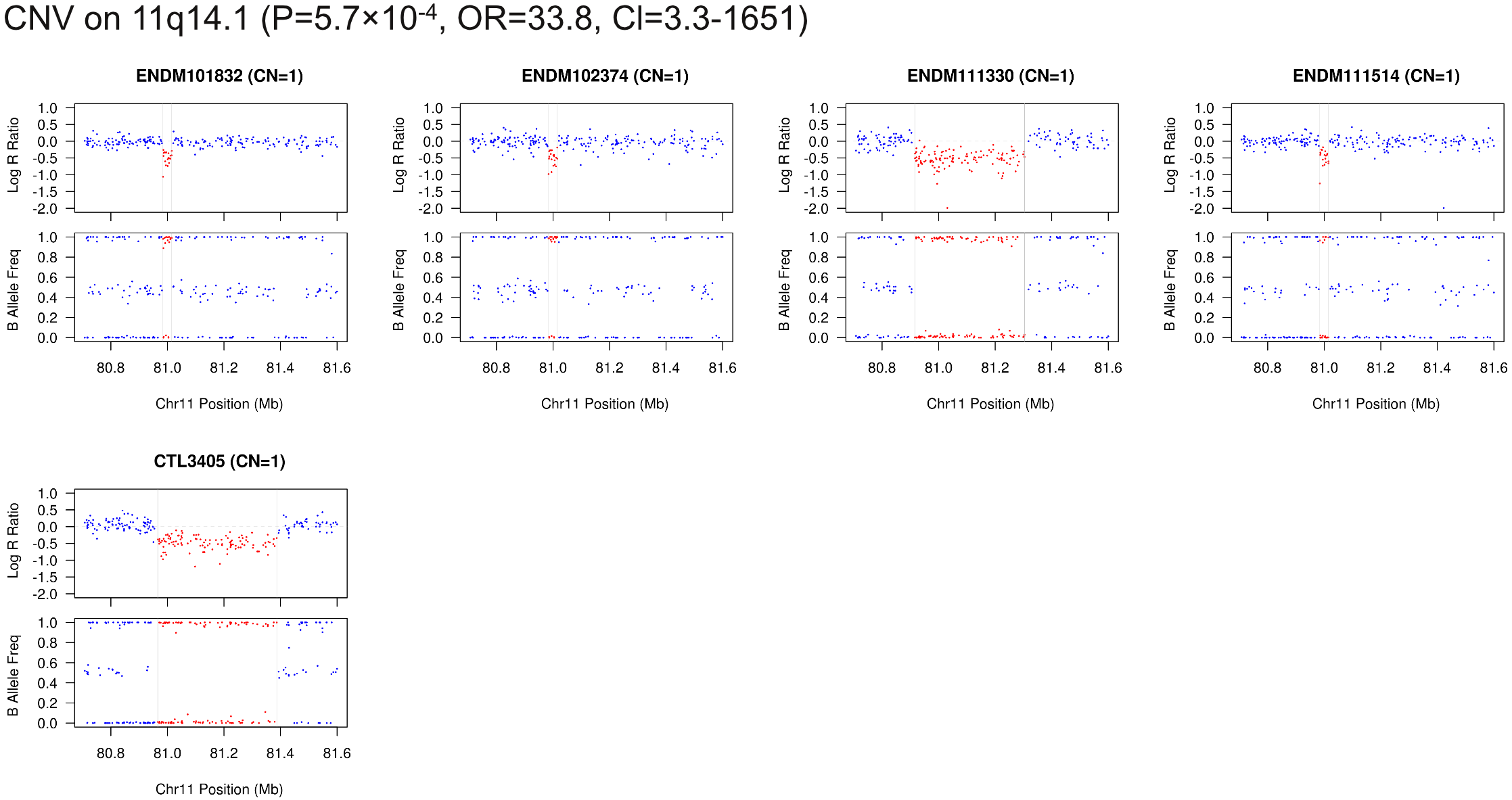


**Figure S5**. The figure shows the LRR and BAF plots of all samples with a CNV at the chromosome 11q14.1 locus. Cases have individual IDs starting with ENDM and population controls have IDs starting with CTL.


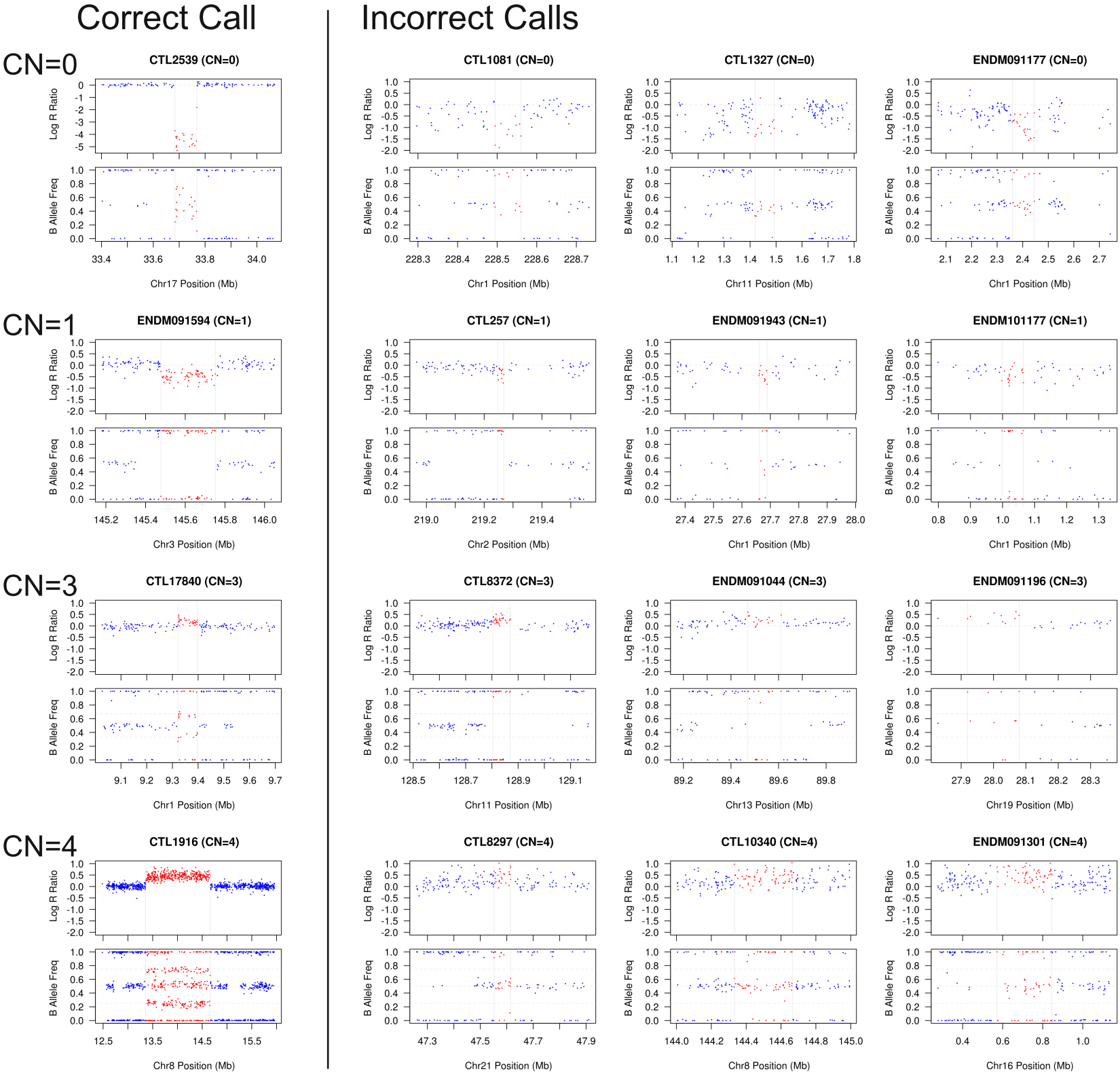


**Figure S6**. The figure shows in the left-side panel the characteristic plots of the four different CNV-states discussed in the paper. Each copy-number state has certain characteristics that define a correct call and, conversely, there are characteristics that are incompatible with a given CN-state (summarized in Table S3 in File S2). The three examples of incorrectly called CN=0 all show inadequate LRR-shift and narrow bands around 0.5 in the BAF panel suggest the samples are heterozygote. The incorrectly called CN=1 show that the LRRs in the CNV regions to be generally similar to the flanking segments and the BAF in the middle sample has signal around 0.5 suggesting the sample is a heterozygote. In the case of the incorrectly called CN=3 examples the LRR show inadequate shift relative to the flanking segments and third of the samples show BAF signal around 0.5 which is incompatible with this state. As for CN=4 the LRR in the CNV regions similar to the flanking segments and none of the BAF patterns has the characteristic 5-band profile.

| **Table S1. Summary of CNVs > 1Mb** | | | | | | |
| --- | --- | --- | --- | --- | --- | --- |
|  | **CNV-count > 1Mb** | | |  | **CNV-length max (bp)** | |
|  | **Deletion** | **Duplication** | **All** |  | **Deletion** | **Duplication** |
| Cases | 15 (0.70%) | 27 (1.27%) | 42 (1.97 %) |  | 6,244,604 | 3,899,583 |
| Controls | 120 (0.67%) | 280 (1.56%) | 400 (2.22%) |  | 4,234,583 | 5,972,672 |
| All | 135 | 307 | 442 |  |  |  |
| Table S1 show the absolute counts of CNVs > 1Mb with the corresponding population frequency reported in parentheses. The results indicate that CNVs > 1Mb represent 1.1% (442 of 38,609) of all CNVs post-filter in the present study, and have a population prevalence of 2.2% (442 of 20,146). There is a slight enrichment of large CNVs in controls compared to cases, indicating a lack of association between large CNVs and endometriosis. Interestingly the proportion between deletions (31%) and duplication (69%) in these large CNVs differ significantly from the overall proportion between deletions (47%) and duplications (53%). One interpretation of this phenomenon could be that large deletions are more detrimental compared to duplications. Large CNVs are frequently reported by PennCNV as multiple adjacent CNVs. This is due to local fluctuations in LRR and BAF which causes such large CNVs (predominantly duplications) to break apart. To assess the true extend of large CNVs we merged adjacent duplications from the same individual that were less than 100kb apart. We found 1419 CNVs that after merging were collapsed into 667 larger CNVs. Only the 442 CNVs > 1Mb are included here. | | | | | | |

| **Table S2. Very rare CNVRs detected in cases only** | | | | | | | | | |
| --- | --- | --- | --- | --- | --- | --- | --- | --- | --- |
| **CNV characteristics** | | | | | | **Statistics** | | **CNV Counts** | |
| **Locus** | **Cytoband** | **Gene** | **Probes** | **base pairs** | **gain/loss** | **P-value** | **OR** | **Case (n=2,126)** | **Control (n=17,974)** |
| chr1:194364761-194422151 | 1q31.3 |  | 11 | 57,391 | loss and gain | 1.24×10^-4^ | Inf | 4 | 0 |
| chr2:206748062-206830070 | 2q33.3 |  | 20 | 82,009 | loss | 1.11×10^-2^ | Inf | 2 | 0 |
| chr3:121968266-121984020 | 3q21.1 | CASR | 11 | 15,755 | gain | 1.11×10^-2^ | Inf | 2 | 0 |
| chr3:137185210-137324323 | 3q22.3 |  | 30 | 139,114 | gain | 1.11×10^-2^ | Inf | 2 | 0 |
| chr6:1649347-1689999 | 6p25.3 | GMDS | 20 | 40,653 | loss | 1.11×10^-2^ | Inf | 2 | 0 |
| chr6:108848121-108867031 | 6q21 | FOXO3 | 10 | 18,911 | gain | 1.11×10^-2^ | Inf | 2 | 0 |
| chr7:94368896-94606271 | 7q21.3 | PPP1R9A | 34 | 237,376 | gain | 1.11×10^-2^ | Inf | 2 | 0 |
| chr9:77349844-77545238 | 9q21.13 | TRPM6 | 44 | 195,395 | gain | 1.11×10^-2^ | Inf | 2 | 0 |
| chr9:92469261-92524437 | 9q22.2 | UNQ6494 | 17 | 55,177 | gain | 1.11×10^-2^ | Inf | 2 | 0 |
| chr11:14690191-14736258 | 11p15.2 | PDE3B | 11 | 46,068 | loss and gain | 1.11×10^-2^ | Inf | 2 | 0 |
| chr18:36981984-37018183 | 18q12.2 | LOC647946 | 8 | 36,199 | loss and gain | 1.11×10^-2^ | Inf | 2 | 0 |
| chr21:32714111-32804036 | 21q22.11 | TIAM1 | 24 | 89,925 | loss and gain | 1.11×10^-2^ | Inf | 2 | 0 |
| chr21:43716900-43746695 | 21q22.3 | ABCG1,TFF3 | 16 | 29,796 | loss and gain | 1.11×10^-2^ | Inf | 2 | 0 |
| The table list 13 CNVRs with at least 2 CNVs found in cases only. The intragenic CNVR on 1q31.3 include three CNVs with loss and one with gain. If all four CNVs have deleterious effects this CNVR also pass the genome-wide threshold for significance. | | | | | | | | | |

| **Table S3. LRR and BAF characteristics of four copy-number states** | | | | | |
| --- | --- | --- | --- | --- | --- |
|  | **LRR** | |  | **BAF** | |
|  | **Correct** | **Incorrect** |  | **Correct** | **Incorrect** |
| CN=0 | Steep (>2) and consistent shift | Modest (<2) and inconsistenet drop |  | Diffuse cloud between 0.2-0.8 | Narrow banding around 0.0, 0.5 and 1.0 |
| CN=1 | Consistent shift in LRR compared to flanking regions | Modest and inconsistenet drop similar to flanking regions. |  | Narrow banding around 0.0 and 1.0 | Banding around 0.5 |
| CN=3 | Consistent shift in LRR compared to flanking regions | Modest and inconsistenet increase similar to flanking regions. |  | Heterozygous duplications has four bands at 0.1, 0.4, 0.6 and 1.0, and homozygous duplication has two bands at 0.0 and 1.0 | Banding around 0.5 |
| CN=4 | Consistent strong shift in LRR compared to flanking regions | Inconsistenet increase similar to flanking regions. |  | Heterozygous duplications has five bands at 0.1, 0.3, 0.5, 0.7 and 1.0, and homozygous duplication has two bands at 0.0 and 1.0 | (blank) |
| Based on the characteristics provided in the table we devised a set of filters to apply to the raw PennCNV CNV calls. After applying these filters a large portion of the incorrectly assigned CNV calls were eliminated. | | | | | |

| **Table S4. Power to detect** | | | | | |
| --- | --- | --- | --- | --- | --- |
|  | **CNV frequency** | | | | |
| **OR** | **0.001** | **0.002** | **0.003** | **0.004** | **0.005** |
| 2.0 | 0.517 | 0.770 | 0.898 | 0.957 | 0.983 |
| 2.5 | 0.774 | 0.959 | 0.994 | 0.999 | 1.000 |
| 3.0 | 0.920 | 0.996 | 1.000 | 1.000 | 1.000 |
| 3.5 | 0.978 | 1.000 | 1.000 | 1.000 | 1.000 |
| 4.0 | 0.995 | 1.000 | 1.000 | 1.000 | 1.000 |
| 4.5 | 0.999 | 1.000 | 1.000 | 1.000 | 1.000 |
| 5.0 | 1.000 | 1.000 | 1.000 | 1.000 | 1.000 |
| The Power-to-Detect CNV association in the present study of 2,126 endometriosis cases and 17,974 population controls is shown given a range of odds-ratios (OR) between 2 and 5 and CNV frequencies ranging between 0.001 and 0.005. | | | | | |
